# Traumatic Brain Injury and Stem Cell: Pathophysiology and Update on Recent Treatment Modalities

**DOI:** 10.1155/2017/6392592

**Published:** 2017-08-09

**Authors:** Cesar Reis, Vadim Gospodarev, Haley Reis, Michael Wilkinson, Josileide Gaio, Camila Araujo, Sheng Chen, John H. Zhang

**Affiliations:** ^1^Department of Physiology and Pharmacology, Loma Linda University School of Medicine, 11041 Campus Street, Risley Hall, Room 219, Loma Linda, CA 92354, USA; ^2^Loma Linda University School of Medicine, Loma Linda, CA 92354, USA; ^3^Department of Epidemiology & Biostatistics, Loma Linda School of Public Health, Loma Linda, CA 92350, USA; ^4^Department of Neurosurgery, Second Affiliated Hospital School of Medicine, Zhejiang University, Hangzhou, China; ^5^Department of Neurosurgery, Loma Linda University School of Medicine, Loma Linda, CA 92354, USA

## Abstract

Traumatic brain injury (TBI) is a complex condition that presents with a wide spectrum of clinical symptoms caused by an initial insult to the brain through an external mechanical force to the skull. In the United States alone, TBI accounts for more than 50,000 deaths per year and is one of the leading causes of mortality among young adults in the developed world. Pathophysiology of TBI is complex and consists of acute and delayed injury. In the acute phase, brain tissue destroyed upon impact includes neurons, glia, and endothelial cells, the latter of which makes up the blood-brain barrier. In the delayed phase, “toxins” released from damaged cells set off cascades in neighboring cells eventually leading to exacerbation of primary injury. As researches further explore pathophysiology and molecular mechanisms underlying this debilitating condition, numerous potential therapeutic strategies, especially those involving stem cells, are emerging to improve recovery and possibly reverse damage. In addition to elucidating the most recent advances in the understanding of TBI pathophysiology, this review explores two primary pathways currently under investigation and are thought to yield the most viable therapeutic approach for treatment of TBI: manipulation of endogenous neural cell response and administration of exogenous stem cell therapy.

## 1. Introduction

Traumatic brain injury (TBI) is a leading cause of death and disability, affecting approximately 1.7 million Americans annually [1]. According to the World Health Organization, TBI will continue to be a major health problem and primary reason for disability leading into 2020 [2]. The proportion of TBI-related hospitalizations due to motor vehicle accidents increases through age 44 before decreasing beginning at ages 45–64, when falls become the leading cause of TBI-related hospitalization [3]. Traumatic brain injury pathophysiology includes blood-brain barrier breakdown, widespread neuroinflammation, diffuse axonal injury, and subsequent neurodegeneration [4]. Several treatment options to date include hyperbaric oxygen therapy, noninvasive brain stimulation, task-oriented functional electrical stimulation, and behavioral therapies [5]. There is an emerging treatment option for brain injury, which entails the use of stem cells for neuroregeneration and repair. Exogenous stem cell transplantation has been shown to increase endogenous cellular proliferation and promote immature neural differentiation in the injured region of the brain [6]. Understanding regenerative capacities of endogenous neural stem cells, as well as the impact of exogenous neural stem cells on proliferation and differentiation, will further elucidate how to improve functional recovery and brain repair after TBI.

## 2. Pathophysiology

In direct contrast to other tissues in the mammalian body, the brain is unable to properly regenerate and reconnect the injured areas to the uninjured areas of the brain [7]. The detailed pathophysiology of traumatic brain injury remains to be fully elucidated, but there are several components that have been studied and widely accepted as normal sequela following brain injury.

**Figure 1:**
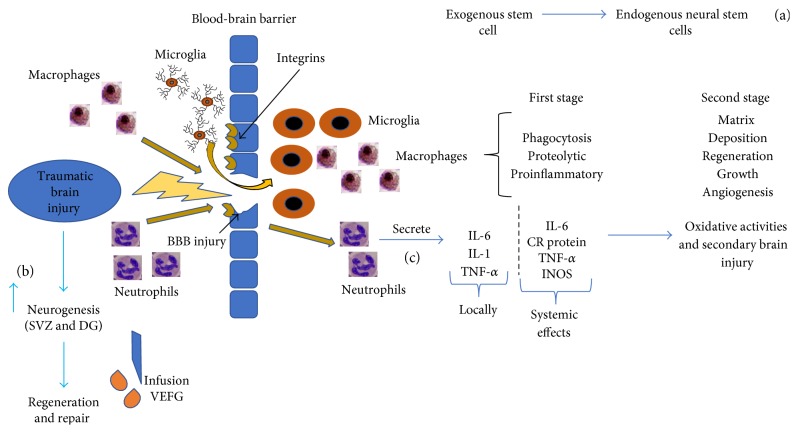
After TBI, exogenous stem stimulate proliferation of endogenous neural stem cells (a). After TBI, there is induction of neurogenesis (b) post-TBI infusion of VEGF. After TBI and rupture of the blood-brain barrier, macrophages will stimulate an initial phase of phagocytic, proteolytic, and proinflammatory functions, while the second phase is characterized by anti-inflammatory functions, which includes regeneration, growth, angiogenesis, and matrix deposition. Microglia initiate inflammatory events. This figure also demonstrates neutrophil invasion and their impact on pathological processes of brain trauma, which includes alteration of vascular permeability and contribution to oxidative damage via secretion of lysosomal enzymes, and changes in cerebral blood flow. In addition, neutrophils act by releasing inflammatory cytokines such as IL-6, IL-1, and tumor necrosis factor alpha (TNF-*α*) (c). Microglia, monocytes, macrophages, and neutrophils invade areas exhibiting blood-brain barrier damage, and there is extensive upregulation of neutrophil adhesion factors including integrin receptors. Gerry Shaw, microglia and neurons, 25 July 2005, by Creative Commons; hematologist, segmented neutrophils, 31 August 2009, Creative Commons; microphages by Patho via Wikimedia Commons; microglia by Frontier in Cellular Neuroscience, 30 January 2013, Creative Commons.

After brain injury, there are blood-brain barrier breakdown and neurodegeneration in areas including the injured cortex, hippocampus, and a portion of the diencephalon. Microglia, monocytes, macrophages, and neutrophils invade areas exhibiting blood-brain barrier damage (Figure 1) [8], which is also associated with extensive upregulation of neutrophil adhesion factors [9], including integrin receptors (Figure 1) and immunoglobulin superfamily members [10–12]. This process is carried out through innate signaling pathways and, in the case of TBI, through release of damage-associated molecular pattern molecules (DAMPs), better known as danger signals [13]. This response is an effort to restore normal homeostasis, but, if the extent of injury is too great, maladaptive immune responses can ensue. This inflammatory response can persist for years and eventually contributes to neurodegeneration. However, the use of anti-inflammatory medications shortly after TBI has not been shown to be an effective treatment, which suggests that inflammation may play a beneficial role, particularly in the acute phase of TBI [14].

## 3. Microglia

Microglia are the first responders in TBI and initiate inflammatory events. These cells have been shown to remain in the injured area more than one year following brain injury. It is unclear if microglia are responding to the degenerative process or are active players in the prolonged white matter degeneration [15]. Studies have revealed that in the acute phase response to cell death, microglia play a neuroprotective role by transforming into highly mobile phagocytic cells as they insert themselves into the damaged glial limitans, connecting to form a phagocytic barrier. Prevention of this response by blocking purinergic receptor signaling or connexin hemichannels results in exacerbation of pathological processes, including increased leaking of material into brain parenchyma [8]. Microglia are capable of different polarization states known as M1 and M2. The M1 macrophage response is rapidly induced and maintained at the site of injury and typically overwhelms the smaller and shorter-lived anti-inflammatory response of M2 cells. TNF-*α* and IFN*γ* promote differentiation into M1, which is capable of producing oxidative metabolites and proinflammatory cytokines. This response is essential for host defense but can lead to secondary damage in healthy cells and tissue [16–18]. M2 cells are activated in the presence of IL-4 [19] or IL-1*β* [20], promote angiogenesis and matrix remodeling, and regulate the immune system. A recent study found that cycle AMP functions synergistically with IL-4 to drive the M1 to M2 conversion after experimental spinal cord injury. The presence of M2-converted microglia ameliorated production of proinflammatory cytokines, including TNF-*α* [21]. Kigerl and colleagues found further evidence supporting M1 macrophages to be neurotoxic and M2 cells to be neuroprotective. Finding a way to shift infiltrating blood monocytes towards the M2 phenotype after TBI may promote repair and regeneration and decrease secondary injury caused by proinflammatory events [22].

The use of stem cells in inducing earlier and longer-lasting effects of M2 was studied using mesenchymal stem cells (MSCs) after TBI. They found upregulation of M2 expression markers in mice 3 and 7 days after TBI, as well as reduction of lysosomal activity in microglia 7 days after TBI. Intracerebroventricular infusion of MSCs promoted proregenerative activity and reduced phagocytosis, effectively reversing the M1 proinflammatory phenotype typically acquired by microglia after brain injury [23]. A study investigating multipotent adult progenitor cells (MAPCs) to modulate the microglia phenotype found a systemic reaction in response to the stem cells, particularly with T regulatory cells in the spleen and blood. In order to increase the M2/M1 ratio and increase M1 macrophage apoptosis using MAPCs, direct contact between the stem cells and splenocytes was required [24]. In the case of MSCs, they act locally in the brain at the lesion site and control the polarization of microglia through the release of active molecules instead of cell-cell contact [23, 25]. A study using MSCs in a mice model found they were able to alter the ratio of IL-10 and TNF-*α* in favor of IL-10, further supporting MSCs' ability to shift microglia to an anti-inflammatory phenotype. In addition, they found enhanced proliferation of T lymphocytes in microglia-MSC cocultures, indicating increased antigen-presenting ability of microglia in the presence of MSCs [26]. Investigating stem cells that can induce early and persistent M2 phenotypes to foster growth and tissue repair is an important topic in stem cell translational research.

## 4. Neutrophils, Monocytes, and Macrophages

Neutrophil invasion has a significant impact on pathological processes of brain trauma, which includes alteration of vascular permeability [27], contribution to oxidative damage via secretion of lysosomal enzymes, and changes in cerebral blood flow. Neutrophils act by releasing inflammatory cytokines such as IL-6, IL-1, and tumor necrosis factor alpha (TNF-*α*) (Figure 1(c)) [28]. Shortly following TBI in human subjects, researchers found a systemic inflammatory response as evidenced by an increase in circulating leukocyte counts, elevating expression of TNF-*α*, IL-6, C-reactive protein, and iNOS. This increase in oxidative activity not only can lead to systemic damage but can further exacerbate secondary local damage at the initial site of TBI [29]. However, since neutrophils can recruit monocyte-derived macrophages, it was interesting to find that when this ability was blocked, mice were found to inadequately repair and recover motor skills, according to a study by Shechter and colleagues [30].

Macrophages exhibit an initial phase of phagocytic, proteolytic, and proinflammatory functions, while the second phase is characterized by anti-inflammatory functions, which include regeneration, growth, angiogenesis, and matrix deposition (Figure 1) [31, 32]. In direct contrast, another study by Hsieh in 2014 demonstrated improved hippocampal neuronal survival and functional recovery by reducing the number of macrophages following cortical injury 2–4 weeks after injury. This study revealed an association between the C-C chemokine receptor 2, which guides monocytes to inflamed tissues, and pathological processes in chronic stages of TBI [33].

## 5. Axonal Degeneration

In addition to ongoing inflammation following TBI, a study of the porcine brain injury model by Chen and colleagues revealed that axonal degeneration continues up to 6 months following initial brain trauma, which causes continued impaired axonal transport and, in effect, accumulation of amyloid precursor proteins (APP) and amyloid-B (A*β*) peptides. These results are very important since A*β* is a hallmark in pathology of Alzheimer's disease, and many studies have linked brain trauma with an increased risk of developing Alzheimer's disease [34, 35]. Thus, accumulation of proteins, particularly in the discrete swellings at the terminal ends of disconnected axons, may be linked to lysis or leakage of swollen axons, causing protein release into surrounding tissue and cerebrospinal fluid. Another possible cause of APP proteolysis is caspase-3 activation through a cleavage process that interrupts normal intracellular processing of APP [36]. Nikolaev and colleagues found that activation of the APP/death receptor 6/caspase 6 apoptotic pathway leads to axonal destruction following the loss of proteins important for neuronal survival, including brain-derived neurotrophic factor, neurotrophin 3, and nerve growth factor (NGF) activation [37]. A study used this pathway to examine whether it represents a common mechanism for axonal degeneration in response to multiple insults. They found that inhibition of APP cleavage prevented axonal degeneration triggered by NGF withdrawal. However, blocking this pathway did not protect against degeneration caused by mechanical or chemical insults [38]. Though the precise mechanism behind APP proteolysis and subsequent accumulation of A*β* peptides following TBI is unclear, long-term formation of A*β* peptides may play a role in the link between a history of brain trauma and increased risk of developing Alzheimer's dementia.

In summary, the role of specific cellular responses that are associated with inflammation and neurodegeneration in pathophysiology of TBI is quite complex [39]. The natural inflammatory response is crucial to foster healing and regeneration following TBI, but the intricate balance between inflammations, which are thought to promote regeneration and maladaptive chronic neuroinflammation, requires further exploration. Currently, it is with these challenges in mind that novel treatment options have been studied to improve outcomes following TBI, particularly those involving the use of stem cell therapy. It is thought that endogenous adult neural stem cells, as well as progenitor cells residing in the neurogenic regions of the brain, may provide regenerative and reparative function to CNS injuries, such as TBI [40]. More specifically, there is an increased neurogenic response following TBI, especially in the subventricular zone (SVZ) and dentate gyrus (DG) of the hippocampus; eliciting this endogenous response could provide improved regeneration and repair following TBI (Figure 1) [41]. Exogenous stem cell therapy is also an area of interest for treatment of TBI, as it is thought to not only provide repair mechanisms but also stimulate proliferation of endogenous neural stem cells [42] (Figure 1).

## 6. Endogenous Neural Stem Cells

Traumatic brain injury places significant stress on the human brain, making it very difficult to maintain appropriate cognitive abilities. Although other organs within the body, such as the skin, possess the capability to self-renew after injury, the brain cannot simply regenerate. Much of the focus within the last 10 years has been spent on discovering the impact of neural stem cells on the regenerative efforts of the brain. Since the 1960s, it has been suggested that new adult brain cells are capable of regenerating; however, it was not until the late 1990s that confocal microscopy revealed that newborn brain cells can differentiate into neurons upon maturation [43, 44].

Neural stem cells have been localized to two regions of the adult brain, namely, the SVZ of the lateral ventricles, which generate neuroblasts that travel via a rostral migratory stream (RMS) to the olfactory bulb and the subgranular zone (SGZ) of the hippocampal DG, which integrate within the DG and become fully mature within a few weeks in a process called adult hippocampal neurogenesis [45]. It remains unclear whether these NSC regions can replace the lost neurons after damage or injury. Most recently, it has been suggested that the neurogenic system within the SVZ of adults is inactive [46]. Although this system has been shown to be present in other animal models such as rodent studies, it remains unclear how much migration or neuroblast development occurs in the olfactory bulb [47]. However, repeated evidence suggests that NSCs are found in the SVZ of adults and extensive migration to the olfactory bulb occurs in infants up to 18 months [48].

There has been some evidence to support self-renewal of progenitor cells in the DG occurring throughout life suggesting that some newly regenerated cells are morphologically and phenotypically similar to hippocampal neurons [49]. Just how much hippocampal self-regeneration occurs and to what functional significance it possesses have led to further investigation. It has been shown that up to one-third of hippocampal cells turn over during adulthood while also exhibiting a 4-fold decline in the amount of neuroblasts resulting in a net loss of hippocampal neurons including the DG despite some degree of neurogenesis. Functionality of hippocampal neurogenesis has not been adequately assessed; however, comparison of neurogenesis in humans with rodent studies may suggest that similar functionality is dependent on similar regeneration rates. Furthermore, because the DG acts as a control mechanism in neuronal circuitry, small amounts of neurogenesis here could have substantial influence on pattern separation in the process of new memory formation [50].

## 7. Response of NSC to TBI

Increasing evidence suggests that TBI induces neurogenesis in animal models via ipsilateral NSC maturation and integration into functionally active brain cells of peridamaged regions of the hippocampus [51]. Interestingly, both human and animal cerebral cortex and regions of white matter exhibit TBI-induced neurogenesis either from proliferation of cells from neurogenic regions such as the SVC or from locally born cells [52, 53]. It is well accepted that neurogenesis is induced following events of TBI (Figure 1), but more recently efforts to quantify recovery from TBI have shown variable results. One animal study revealed that cognitive recovery does occur in rats [54]. In humans, several adult brain maladies have been shown to induce neurogenesis, such as that associated with Huntington's disease [55], ischemic stroke [56], Alzheimer's disease [57], epilepsy [58], and hemorrhage [59]. Neurogenesis following TBI has been studied in humans to see whether newborn cells have the capacity to replace those that are damaged, thereby restoring brain function. Recent studies revealed cognitive deficit recovery in human models following TBI with increased recovery of cognitive function seen in children compared to that in adults [54, 60].

## 8. Manipulation of NSC in Response to TBI

The regenerative capacity of NSCs found in the adult brain is important for responding to TBI-related injuries. Recently, manipulation of various growth factors has shown significant efficacy in promotion of neurogenesis. In adult animal studies, it has been shown that intraventricular infusion of basic fibroblast growth factor (bFGF) and epidermal growth factor (EGF) enhances cell proliferation in the hippocampus and SVZ leading to improved cognitive function [61, 62]. In addition, post-TBI infusion of recombinant VEGF improved neurogenesis in the SVZ, thereby promoting recovery and aid in survival of neurons produced in the DG (Figure 1) [63, 64]. Besides growth factors, pharmaceutical agents like statins [65], erythropoietin [66], and even antidepressants, such as imipramine [67], have shown to enhance endogenous neurogenesis and lead to improved cognitive recovery.

Compared to animal brain models, simply promoting neurogenesis is significantly more complex in humans. Concerns over possible treatment-related injuries from stimulation of endogenous neurogenesis following TBI are still under investigation and are associated with worrisome results. It has been suggested that post-TBI–induced neurogenesis has contributed to the onset of post-TBI epilepsy [68]. Specifically, increased neurogenesis within the hippocampus has been known to result in development of epilepsy and other seizure-like symptoms. However, ablation of aberrant seizure-induced hippocampal neurogenesis can reduce incidence of seizures and essentially cure epilepsy. The technical challenges surrounding human models in manipulating NSC in response to TBI remain a burgeoning area of focus [69].

## 9. Exogenous Stem Cells

### 9.1. Embryonic Stem Cells

The use of embryonic stem cells (ESCs) in the treatment of traumatic brain injury remains a growing field of research. Three notable clinical trials have used embryonic stem cells in TBI rodent models with promising results. Across the board, using ESCs is associated with better outcomes, such as recovery of motor function [70], improved cognitive function, and high survival of transplanted cells [71].

Ikeda and colleagues were one of the first investigators to use nonhuman primate stem cells, which they used to study embryonic stem cells' capacity to restore function to damaged neural tissue. In this experiment, Cynomolgus monkey embryonic stem cells were first treated with retinoic acid before being transplanted into the periventricular area of mice brains. Some of the treated cells later developed into Islet1+ motoneurons. These cells were then transplanted into mice that had undergone an experimental stroke model of brain injury. Approximately one month after the transplant, mice in the experimental arm had greater recovery of motor function than that in the control mice [70].

The team of Peruzzaro and colleagues aimed to determine if an enriched postsurgical environment will have a beneficial effect on three key factors of embryonic stem cell transplants in terms of their integration, migration, and survival. In a rodent TBI model, the medial frontal cortex was injured via cortical impact. One group of rats was placed into an enriched environment, while the other was placed in a standard environment. In each group, two arms of the study emerged; the rats were treated with either murine cortical embryonic stem cells or did not receive such treatment. The enriched environment animals performed statistically as well as the sham group, compared to groups with just embryonic stem cells or only an enriched environment [72]. This trial highlighted the importance of an enriched postsurgical environment in the healing process. Although the enriched environment groups were not outperforming the other groups by a statistically significant measure, these results are promising because they represent building blocks for future studies. Further research could incorporate the use of enriched environments in stem cell and TBI studies to investigate this phenomenon.

A preclinical trial performed by Haus and colleagues aimed to study the long-term benefits of treating TBI with neural stem cells. Rats underwent an impact immunodeficient model of TBI and were found to demonstrate hippocampal-dependent spatial memory impairment for at least two months. The experimental group, treated with transplanted human neural stem cells, had better long-term consequences, including an increased survival in the host hippocampal cells, improved cognitive function, and no change in scar tissue. The investigators concluded that although no change was observed in the volume of scar tissue, beneficial effects were still seen, suggesting that measure of recovery should focus more on percentage of surviving cells rather than a decrease in lesion volume. This study also observed a small number of improved behavioral components correlating with a 9–25% survival rate of the transplanted cells [71]. This correlation of cell survival with improvement in behavioral measures is an encouraging revelation, which provides a foundation and direction for future studies.

### 9.2. Multipotent Adult Progenitor Cells

MAPCs are a population of bone marrow-derived adherent progenitor cells [73]. First isolated in 2002, MAPCs are able to differentiate in vitro into MSCs but also cells with visceral mesoderm, neuroectoderm, and endoderm characteristics and proliferate extensively with little loss of differentiation potential or senescence [74]. MAPCs can be considered a distinct in vitro cell population from MSCs, as they express different surface proteins, have lower MHC Class I expression, and display more robust endothelial differentiation [75]. Bedi and colleagues investigated the long-term effects of MAPC treatment after TBI on microglia phenotype as well as cognitive and motor function. They found a localized reduction in activated microglia 120 days after injury in the DG of the hippocampus, contributing to reduction in the prolonged neuroinflammatory response and preservation of normal neuronal responses. MAPC therapy improved spatial learning, information retention, and memory retrieval as well as motor deficits, demonstrating the utility of intravenous administration of MAPCs to improve long-term cognitive function following TBI [76].

### 9.3. Adult Neural Stem Cells

Neural stem cells (NSCs) are multipotent cells that can differentiate into neural cells; however, their differentiation into other tissue types is limited [77]. NSCs are found in the SVZ of the lateral ventricle and the SGZ of the hippocampal dentate gyrus, as well as other parts on the brain, such as cerebral cortex, amygdala, hypothalamus, and substantia nigra. These cells can be isolated, grown in culture, and generate multiple neural lineages, which can be used in neurological disorders as an essential component of cell-replacement therapy [78].

In a TBI rat model, adult NSCs were transplanted into injured areas of the brain. They survived the transplantation process and migrated to injured sites while expressing markers for mature astrocytes and oligodendrocytes [79]. In other rat model studies, two weeks after the NSCs were transplanted in the cortex, it was revealed that approximately 1-2% of the cells became engrafted and were able to improve motor function [80]. Park and colleagues reported that injured rats experienced an improved cognitive function after NCS were transplanted into the hippocampal region [81]. Moreover, Lee et al. revealed that hemorrhagic stroke model rats, when injected with NCSs intravenously or intracerebrally, had a decrease in the initial neurologic deterioration and less edema due to the anti-inflammatory and antiapoptotic properties of NSCs [82].

The optimal time window for transplantation is 7–14 days [77], and beyond that, the glial scar forms, inhibiting perfusion and graft survival [83]. A significant challenge with NSC transplantation is the ability to deliver cells to the area of interest. Routes of administration include intrathecal, intravenous, and intra-arterial infusion. Unfortunately, the engraftment rates are low and there is a constant risk for embolus formation during intravascular infusions. However, a nanofiber scaffold implantation was suggested by Walker et al. as a novel method to be used to provide the support necessary for cell proliferation, which gives direction to future studies [84].

### 9.4. Inducible Pluripotent Stem Cells

To observe functional aspects of transplanted induced pluripotent stem cells (iPSCs) compared to those of ESCs, Wang and colleagues used a rodent model of ischemia and three different treatment options, which were comprised of pluripotent stem cells, embryonic stem cells, and phosphate-buffered saline for the control. The animals received injections into the left lateral ventricle stereotactically. At the two-week period, it was found that the embryonic stem cell treatment group animals exhibited a marked recovery in their glucose metabolism, which was then followed by a decrease. Imaging tests were performed approximately one month after treatment. Both stem cell treatment groups had better neurologic scores than the control group, signifying that the experimental groups experienced greater recovery of their cognitive function. Further analysis revealed that the transplanted cells survived and migrated to the region of ischemia. However, in the conclusion of their study, the investigators endorsed that iPSCs may be a preferable option to ESCs [85].

In a similar study conducted by Perruzzano, the team of Dunkerson and colleagues explored the impact of an enriched environment in stem cell transplants. Since treatment with iPSC therapy had previously produced promising results, it was time to evaluate the efficacy of the two therapies combined. An animal TBI model of impact to the medial frontal cortex was used. As in previously mentioned experiments, two groups were formed: one in an enhanced environment and the other in a standard environment. Approximately one week following injury, the rats underwent either an iPSC transplant or media control. Notably, the rats that were exposed to both an enriched environment and iPSCs performed as well as the sham and enriched environment group. Across the board, the combined therapy group demonstrated the best cognitive recovery in comparison to that of other groups. Investigators concluded that combined therapy must be strongly considered in future studies that attempt to explore treatment options for TBI [86].

Although iPSC therapy is promising, like many treatments, it is not free of potential side effects. Early animal model studies of iPSCs showed a tendency for embryonic-derived cells to form benign tumors from the embryonic germ layers. Some of the cells transplanted into animal models have led the animals to alternatively develop conditions such as neurodegenerative disease. However, assays have been developed to target problem cells and decrease these events [87]. One of such assays encourages the differentiation of iPSCs into MSCs (iPSCs-MSCs). The traditional protocol used for differentiation of human iPSCs was modified by initially inhibiting Smad2/3 signaling in iPSCs cultured with mTeSR1 medium followed by passaging cells by trypsinization in 7.5% CO_2_ using plastic culture dishes to improve the differentiation into MSCs. The modified protocol achieved enrichment of iPSCs-MSCs, as they expanded more rapidly and to a greater extent, but eventually underwent senescence. This method did not result in teratomas in murine models [88]. Another tactic for increasing the safety of iPSCs includes the addition of inducible caspase-9 (iC-9) suicide gene. An in vitro mouse model demonstrated the possibility of inducing apoptosis in tumors grown from iPSC transplants without interfering with the differentiation of the stem cells into neurons [89]. An in vivo study has similarly used caspase-9 to decrease tumor size. Following a subcutaneous transplantation of iPSCs with iC-9, teratoma formation occurred. A chemical inducer of dimerization was then administered, and the teratomas had drastically reduced [90]. Since using this gene has no detrimental effects on the differentiation of the iPSCs into neurons, it seems like a promising treatment for use in TBI.

### 9.5. Mesenchymal Stem Cells

MSCs are multipotent stromal cells derived from a variety of tissues [91] and have the capacity to differentiate into mesenchymal and nonmesenchymal tissue, including neural cells [92]. The ease of access and abundance of sources, as well as their potential to differentiate, have brought these cells to attention of investigators conducting studies in regenerative medicine.

The ability of MSCs to differentiate into neural cells was evidenced by Sanchez-Ramos and colleagues. They demonstrated that when human and mouse MSCs are exposed to specific experimental culture conditions, human and mouse MSCs have the ability to differentiate into neuron and glia-like cells [92]. In addition, MSCs have also been shown to cause an increase in proliferation and differentiation of native NSCs; the mechanism of which may be directly related to chemokines released by MSCs or indirectly via activation of surrounding astrocytes [93].

Besides their ability to differentiate, MSCs selectively migrate to injured tissues in TBI rat models, with subsequent differentiation in neurons and astrocytes and subsequent improvement in motor function [94]. The proposed mechanism through which this occurs is once again related to chemokines, growth factors [95], and adhesion molecules, such as the vascular cell adhesion molecule (VCAM-1), which allows MSCs to adhere to the endothelium of injured tissue [96]. Zhang et al. investigated the anti-inflammatory and immunomodulatory characteristics of MSCs using a TBI rat model. Neurological function was improved in the MSC group from days 3–28 compared to that in controls. MSC treatment group also had a significant decrease in brain water content, to the point where there was no significant difference between MSC and sham group 72 hours after TBI. MSC treatment reduced the number of microglia/macrophages, neutrophils, CD3 lymphocytes, and apoptotic cells in the injured cortex, as well as proinflammatory cytokines [97].

Another noteworthy fact regarding MSCs is their ability to suppress lymphocytes. This characteristic was observed in vitro and in vivo models. A study conducted by Bartholomew and colleagues made it evident that MSCs reduced the proliferative response of lymphocytes [98], which could be useful in reducing the secondary effects of injury in TBI [99]. MSCs have also been noted to upregulate the expression of TIMP3, a metalloprotease inhibitor, which decreases the permeability of BBB in TBI mouse models, thereby enhancing their ability to recover from TBI [100]. Using MSCs, Li et al. detected significantly reduced areas of hypoperfusion in both remote regions and regions adjacent to brain lesions in TBI rat model. Since perfusion is linked to functional deficits, the authors found that this reduction in hypoperfusion was associated with a greater functional recovery as demonstrated by the modified neurological severity score (mNSS score) [101].

One appeal for use of MSCs as a TBI treatment method is their ability to cross the BBB through paracellular pathways [102]. Given that MSCs can migrate across the BBB and the endothelial cell layers of injured tissue, route of administration can be intravenous, as demonstrated in rats that were intravenously transplanted with MSCs following induced cerebral ischemia or received MSCs directly via injection into the brain lesion [103]. Moreover, research using genetically modified MSCs solely for production of growth factors, cytokines, and chemokines that enhance neuronal cells after injury highlights a novel possibility of TBI treatment without actual MSC transplantation [104].

MSCs provide an opportunity for clinical translation, as evidenced by recent clinical trials. A significant challenge with use of MSCs for TBI treatment remains to be the long-term possibility of brain tumor development due to the MSC's capacity of antitumor response suppression [105]. Two clinical trials aimed to test the feasibility and safety of using MSCs in patients with TBI. In 2008, seven TBI patients received a MSC transplant during a cranial operation and then received a second dose administered intravenously. At the six-month follow-up, patients had improved neurological function with no signs of toxicity [106]. Limitations of this study include a small sample size and lack of a control group.

From 2012-2013, 10 patients with severe TBI were recruited for a phase I clinical trial. MSCs were administered intravenously or intrathecally. Individuals had improvement in neurological function as measured by the NIHSS (National Institutes of Health Stroke Scale), GCS (Glasgow Coma Scale), and GOS (Glasgow Outcome Scale). No mortality or adverse events occurred, bringing support for the safety and feasibility of this treatment. Similar to the previous study mentioned, limitations include a small sample size as well as no control group [107]. Although MSC therapy for TBI appears to be safe and feasible, continued research is needed to better assess the efficacy of treatment compared to controls.

### 9.6. Bone Marrow Stromal Cells

Human bone marrow contains hematopoietic and nonhematopoietic stem cells with multipotent characteristics. Bone marrow stromal cells (BMSCs) differentiate into mesenchymal stem cells and, which when exposed to appropriate conditions, possess capacity to differentiate into numerous cell types [108], such as neuron and glial-like cells [92].

Shen et al. investigated outcomes in TBI rat models that were transplanted with BMSCs. The cultured BMSCs were implanted into the injured area of the brain followed by evaluation of neurologic function. Data analysis revealed an increase in expression of glial cell line-derived neurotrophic factor (GDNF), which is thought to be a potent promoter of neuronal survival, as well as genes of other neurotrophic factors. The investigators also noted that a great number of BMSCs that survived and migrated around the site of injury had done so 14 days following transplantation. Furthermore, the TBI rats with transplanted BMSCs presented with less apoptotic cells when compared to those of the control group and had improved neurologic outcomes [109].

In 2016, Cox et al. conducted an investigation utilizing BMSC treatment in human adults with TBI. This trial enrolled patients that were admitted to trauma or neurotrauma ICU and were assigned to one of the dosage treatment arms. The investigators were able to demonstrate the safety of this treatment modality and downregulation of inflammatory cytokines, as well as its ability to preserve critical regions of the brain, which correlate with an increase in functional outcomes [110].

Local and intravenous administration of BMSCs has been investigated for treatment of neurological injury and other neurological diseases [111]. A study protocol by Weiss et al. proposed to use intranasal tissue as a route of BMSC administration to the CNS through the trigeminal nerve. In their initial results, a Parkinson patient reported improvement in many sensory, as well as motor abilities. The authors anticipate at least a 10% improvement in neurological function [112].

## 10. Stem Cell-Derived Exosomes

MSCs have an important role in improving functional outcome after experimentally induced traumatic brain injury (TBI). Exosomes, which are of endosomal origin, are secreted by all cells, including MSCs [113]. These microvesicles are currently being investigated as another potential therapeutic agent for treatment of TBI. Utilization of exosomes for TBI grew from studies that revealed an improvement in poststroke neuroregeneration, functional recovery, and neurovascular plasticity in animal models [114, 115]. Exosomes are thought to function as vesicular carriers to promote intercellular communication, specifically through transfer of microRNA (miRNA), which is one of the mechanisms that Xin and colleagues revealed to be responsible for neurite outgrowth [116].

In 2015, Zhang and colleagues administered MSC-derived exosomes to TBI rats via tail vein injections. Compared to saline-treated controls, exosome-treated TBI rats exhibited significant functional recovery as well as an increase in newly formed endothelial cells in both the lesion boundary zone and the dentate gyrus. The mechanisms involved may be related to an increase in brain vascular density and angiogenesis in those that received MSC exosome administration. Additionally, exosome treatment significantly reduced brain inflammation by reducing the number of CD68+ microglia/macrophages and GFAP+ astrocytes [117]. As a follow-up, in their most recent investigation, Zhang and colleagues intravenously introduced exosomes generated from MSCs that were cultured in 2-dimensional (2D) versus 3-dimentional (3D) collagen scaffolds into experimentally induced TBI rats, which revealed that exosomes derived from 3D scaffolds were associated with a better outcome, in terms of spatial learning, compared to exosomes derived from MSCs that were cultured in traditional, 2D conditions [118]. The aforementioned investigations reveal a potential mechanism through which MSCs have an important role in improving functional outcome after TBI, namely, through exosomes, and that these microvesicles could hold the key to a more refined therapy for TBI and other neurologically devastating conditions, such as stroke.

## 11. Systemic Anti-Inflammatory and Immune Responses

Important areas to consider when working towards clinical translation of stem cell therapy are choosing which type of stem cells to use and the systemic effects of stem cell therapy. MSCs have been found to reduce the persistent inflammatory response following TBI by decreasing lymphocytes [75]. A study investigating MSCs and how they regulate macrophages found that by secreting secretin tumor cell line-1 (STC-1), MSCs can inhibit inflammasome activation in macrophages and prevent maturation and secretion of proinflammatory cytokines, including IL-1*β* [119]. Kim and colleagues investigated extracellular vesicles (EVs) produced by BM-MSCs as an effective therapy for TBI. The EVs were able to decrease inflammation 12 hours after TBI and improve pattern separation and spatial learning impairments 1 month later [120]. Additionally, the authors note that a limitation to using BM-MSCs for producing EVs is they senesce after undergoing expansion in culture [121], but in preparing this experiment, researchers used a standardized protocol during cell expansion in culture to reduce variation and retain progenitor features and preselected a preparation that could expand well beyond the amount required for experimentation [120, 122]. Understanding how to improve efficacy with cell expansion and differentiation contributes to the effectiveness of using stem cells to promote recovery following TBI.

As mentioned previously, Zhao and colleagues found that iPSC-MSCs were less tumorigenic than BM-MSCs, readily expandable, and homogenous, thereby offering more uniform biological activities [88]. In response, Yun and colleagues investigated the anti-inflammatory effects of iPSC-MSCs in the cornea after chemical and mechanical injury. iPSC-MSCs were able to reduce corneal inflammation, thereby offering an alternative therapy for inflammatory diseases [123].

The proinflammatory environment leads to BBB breakdown and worsens neurological deficits following injury. MAPCs have been shown to bypass the pulmonary capillary bed compared to larger MSCs after intravenous injection, leading to more cells contacting splenocytes [124]. Investigator found that MAPC therapy preserved splenic mass and attenuated BBB permeability secondary to their interaction with splenocytes. MAPCs increased the proliferative rate of CD4+ T cells, IL-4, and IL-10 in stimulated splenocytes and stabilized the vascular environment in the perilesional area [125]. Targeting the inflammatory response following acute TBI is an important aspect of stem cell therapy, as a majority of neurological deficits following TBI is caused by both the initial insult and secondary inflammatory responses. MSCs and utilization of exosomes are indicated for clinical translation, as animal studies continue to support their ability to modulate inflammation and promote neuroregeneration.

## 12. Clinical Translation in Diffuse Axonal Injury

Direct implantation of stem cells promotes neuronal regeneration, improved neurological scores, and anti-inflammatory effects in animal models studying responses after controlled cortical impact injury. However, diffuse axonal injury has not been as thoroughly studied in animal models and present in humans from blast injuries [126] and even mild head injuries [127]. Xu and colleagues used an impact acceleration rat model to study the effects of human-derived oligodendrocyte progenitor cells (hOPCs) from a human ESC line in remodeling myelin and axonal regeneration following diffuse axonal injury. hOPCs were able to survive in the deep sensorimotor cortex and migrate with near exclusive affinity to white matter tracts. In the area surrounding the transplantation site, the percentage of myelin basic protein (+) oligodendrocytes, those that ensheath axons, was significantly higher at 3 months compared to that at 6 weeks and compared to that of shams. This supports not only the notion that human ESCs and NSCs can be guided to specific fates after transplantation but also, because rapid proliferation was not observed, there is a lower possibility of overgrowth or tumors. This study provides a TBI model that targets myelin remodeling as a regenerative strategy following diffuse axonal injury [128].

## 13. Conclusion

Clinical trials have shown that MSC transplantation may decrease TBI patients' sequela and has the potential to become an effective treatment modality [129]. MSC safety and efficacy have been investigated in patients with complications following TBI [130] and determined that earlier interventions lend themselves to better results.

Stem cells used as a marker for TBI recovery and improved Glasgow Coma Scale are the focus of another ongoing investigation. Endothelial progenitor cells, which play an active role in vascular repair and revascularization, have been shown to increase 48 hours after TBI and were correlated to clinical outcomes. In the same study, patients with low circulatory levels of endothelial progenitor cells were more likely to present with poor clinical outcomes [131]. Even though long-term follow-up is pending, utilization of endothelial progenitor stem cell transplantation is another potential therapeutic strategy for future interventions, which may enhance vascular repair in patients with TBI and contribute to an improvement in their neurologic outcome.

Although significant research has been conducted in the area of traumatic brain injury, both in terms of complexity underlying the pathophysiology and utilization of stem cell therapy in its treatment, a lot remains to be understood in order to determine the best method to promote recovery of functional brain tissue. Unfortunately, compared to other mammalian tissues, the brain does not have sufficient capacity to regenerate itself and theoretically requires assistance to do so after TBI. Use of neural stem cell therapy, whether through manipulation of endogenous or transplantation of exogenous NSCs, is an approach that numerous studies revealed to have significant potential to promote recovery of brain function in individuals suffering from TBI-associated disability. However, significant amount of research remains to be done in use of stem cells for treatment of TBI due to our limited understanding of potential complications, unexplored ethical implications, routes of administration, and use of combination/cotransplantation therapy. Combination and cotransplantation therapy that utilize NSCs and other cells, such as astrocytes and endothelial cells, which make the central nervous system's microenvironment more optimal for NSC grafting, likely hold the key to the best approach for treatment of aftermath of TBI, especially when considering the multifaceted nature of its underlying pathophysiology. In summary, all studies and future interventions that may impact treatment of TBI require multicenter and randomized prospective trials with long-term follow-up; this will allow future investigators to define the role and impact that each treatment may have on a given patient population.

## References

[1] Laker S. R. (2011). Epidemiology of concussion and mild traumatic brain injury. *PM R*.

[2] Hyder A. A., Wunderlich C. A., Puvanachandra P., Gururaj G., Kobusingye O. C. (2007). The impact of traumatic brain injuries: a global perspective. *NeuroRehabilitation*.

[3] Coronado V. G., McGuire L. C., Sarmiento K. (2012). Trends in traumatic brain injury in the U.S. and the public health response: 1995-2009. *Journal of Safety Research*.

[4] Xiong Y., Mahmood A., Lu D. (2008). Histological and functional outcomes after traumatic brain injury in mice null for the erythropoietin receptor in the central nervous system. *Brain Research*.

[5] Dang B., Chen W., He W., Chen G. (2017). Rehabilitation treatment and progress of traumatic brain injury dysfunction. *Neural Plasticity*.

[6] Tajiri N., Kaneko Y., Shinozuka K. (2013). Stem cell recruitment of newly formed host cells via a successful seduction? Filling the gap between neurogenic niche and injured brain site. *PLoS One*.

[7] Gage F. H., Temple S. (2013). Neural stem cells: generating and regenerating the brain. *Neuron*.

[8] Corps K. N., Roth T. L., McGavern D. B. (2015). Inflammation and neuroprotection in traumatic brain injury. *JAMA Neurology*.

[9] Soares H. D., Hicks R. R., Smith D., McIntosh T. K. (1995). Inflammatory leukocytic recruitment and diffuse neuronal degeneration are separate pathological processes resulting from traumatic brain injury. *The Journal of Neuroscience*.

[10] Albelda S. M., Smith C. W., Ward P. A. (1994). Adhesion molecules and inflammatory injury. *The FASEB Journal*.

[11] Kochanek P. M., Hallenbeck J. M. (1992). Polymorphonuclear leukocytes and monocytes/macrophages in the pathogenesis of cerebral ischemia and stroke. *Stroke*.

[12] Sloan D. J., Wood M. J., Charlton H. M. (1992). Leucocyte recruitment and inflammation in the CNS. *Trends in Neurosciences*.

[13] Bianchi M. E. (2007). DAMPs, PAMPs and alarmins: all we need to know about danger. *Journal of Leukocyte Biology*.

[14] Russo M. V., McGavern D. B. (2016). Inflammatory neuroprotection following traumatic brain injury. *Science*.

[15] Johnson V. E., Stewart J. E., Begbie F. D., Trojanowski J. Q., Smith D. H., Stewart W. (2013). Inflammation and white matter degeneration persist for years after a single traumatic brain injury. *Brain*.

[16] Longhi L., Perego C., Ortolano F. (2013). Tumor necrosis factor in traumatic brain injury: effects of genetic deletion of p55 or p75 receptor. *Journal of Cerebral Blood Flow and Metabolism*.

[17] Wang C. X., Nuttin B., Heremans H., Dom R., Gybels J. (1996). Production of tumor necrosis factor in spinal cord following traumatic injury in rats. *Journal of Neuroimmunology*.

[18] Ding A. H., Nathan C. F., Stuehr D. J. (1988). Release of reactive nitrogen intermediates and reactive oxygen intermediates from mouse peritoneal macrophages. Comparison of activating cytokines and evidence for independent production. *Journal of Immunology*.

[19] Butovsky O., Bukshpan S., Kunis G., Jung S., Schwartz M. (2007). Microglia can be induced by IFN-gamma or IL-4 to express neural or dendritic-like markers. *Molecular and Cellular Neurosciences*.

[20] Sato A., Ohtaki H., Tsumuraya T. (2012). Interleukin-1 participates in the classical and alternative activation of microglia/macrophages after spinal cord injury. *Journal of Neuroinflammation*.

[21] Ghosh M., Xu Y., Pearse D. D. (2016). Cyclic AMP is a key regulator of M1 to M2a phenotypic conversion of microglia in the presence of Th2 cytokines. *Journal of Neuroinflammation*.

[22] Kigerl K. A., Gensel J. C., Ankeny D. P., Alexander J. K., Donnelly D. J., Popovich P. G. (2009). Identification of two distinct macrophage subsets with divergent effects causing either neurotoxicity or regeneration in the injured mouse spinal cord. *The Journal of Neuroscience*.

[23] Zanier E. R., Pischiutta F., Riganti L. (2014). Bone marrow mesenchymal stromal cells drive protective M2 microglia polarization after brain trauma. *Neurotherapeutics*.

[24] Walker P. A., Bedi S. S., Shah S. K. (2012). Intravenous multipotent adult progenitor cell therapy after traumatic brain injury: modulation of the resident microglia population. *Journal of Neuroinflammation*.

[25] Zanier E. R., Montinaro M., Vigano M. (2011). Human umbilical cord blood mesenchymal stem cells protect mice brain after trauma. *Critical Care Medicine*.

[26] Hegyi B., Kornyei Z., Ferenczi S. (2014). Regulation of mouse microglia activation and effector functions by bone marrow-derived mesenchymal stem cells. *Stem Cells and Development*.

[27] Wedmore C. V., Williams T. J. (1981). Control of vascular permeability by polymorphonuclear leukocytes in inflammation. *Nature*.

[28] Taupin V., Toulmond S., Serrano A., Benavides J., Zavala F. (1993). Increase in IL-6, IL-1 and TNF levels in rat brain following traumatic lesion. Influence of pre- and post-traumatic treatment with Ro5 4864, a peripheral-type (p site) benzodiazepine ligand. *Journal of Neuroimmunology*.

[29] Liao Y., Liu P., Guo F., Zhang Z. Y., Zhang Z. (2013). Oxidative burst of circulating neutrophils following traumatic brain injury in human. *PloS One*.

[30] Shechter R., Miller O., Yovel G. (2013). Recruitment of beneficial M2 macrophages to injured spinal cord is orchestrated by remote brain choroid plexus. *Immunity*.

[31] Nahrendorf M., Swirski F. K., Aikawa E. (2007). The healing myocardium sequentially mobilizes two monocyte subsets with divergent and complementary functions. *The Journal of Experimental Medicine*.

[32] Arnold L., Henry A., Poron F. (2007). Inflammatory monocytes recruited after skeletal muscle injury switch into antiinflammatory macrophages to support myogenesis. *The Journal of Experimental Medicine*.

[33] Hsieh C. L., Niemi E. C., Wang S. H. (2014). CCR2 deficiency impairs macrophage infiltration and improves cognitive function after traumatic brain injury. *Journal of Neurotrauma*.

[34] Jellinger K. A., Paulus W., Wrocklage C., Litvan I. (2001). Effects of closed traumatic brain injury and genetic factors on the development of Alzheimer’s disease. *European Journal of Neurology*.

[35] Lye T. C., Shores E. A. (2000). Traumatic brain injury as a risk factor for Alzheimer’s disease: a review. *Neuropsychology Review*.

[36] Gervais F. G., Xu D., Robertson G. S. (1999). Involvement of caspases in proteolytic cleavage of Alzheimer’s amyloid-beta precursor protein and amyloidogenic A beta peptide formation. *Cell*.

[37] Nikolaev A., McLaughlin T., O'Leary D. D., Tessier-Lavigne M. (2009). APP binds DR6 to trigger axon pruning and neuron death via distinct caspases. *Nature*.

[38] Vohra B. P., Sasaki Y., Miller B. R., Chang J., DiAntonio A., Milbrandt J. (2010). Amyloid precursor protein cleavage-dependent and -independent axonal degeneration programs share a common nicotinamide mononucleotide adenylyltransferase 1-sensitive pathway. *The Journal of Neuroscience*.

[39] Perry V. H., Nicoll J. A., Holmes C. (2010). Microglia in neurodegenerative disease. *Nature Reviews. Neurology*.

[40] Rolfe A., Sun D., Kobeissy F. H. (2015). Stem cell therapy in brain trauma: implications for repair and regeneration of injured brain in experimental TBI models. *Brain Neurotrauma: Molecular, Neuropsychological, and Rehabilitation Aspects*.

[41] Patel K., Sun D. (1640). Strategies targeting endogenous neurogenic cell response to improve recovery following traumatic brain injury. *Brain Research*.

[42] Ahmed A. I., Gajavelli S., Spurlock M. S., Chieng L. O., Bullock M. R. (2016). Stem cells for therapy in TBI. *Journal of the Royal Army Medical Corps*.

[43] Kuhn H. G., Dickinson-Anson H., Gage F. H. (1996). Neurogenesis in the dentate gyrus of the adult rat: age-related decrease of neuronal progenitor proliferation. *The Journal of Neuroscience*.

[44] Altman J., Das G. D. (1965). Autoradiographic and histological evidence of postnatal hippocampal neurogenesis in rats. *The Journal of Comparative Neurology*.

[45] Gage F. H., Kempermann G., Palmer T. D., Peterson D. A., Ray J. (1998). Multipotent progenitor cells in the adult dentate gyrus. *Journal of Neurobiology*.

[46] Bergmann O., Liebl J., Bernard S. (2012). The age of olfactory bulb neurons in humans. *Neuron*.

[47] Curtis M. A., Kam M., Nannmark U. (2007). Human neuroblasts migrate to the olfactory bulb via a lateral ventricular extension. *Science*.

[48] Sanai N., Nguyen T., Ihrie R. A. (2011). Corridors of migrating neurons in the human brain and their decline during infancy. *Nature*.

[49] Eriksson P. S., Perfilieva E., Bjork-Eriksson T. (1998). Neurogenesis in the adult human hippocampus. *Nature Medicine*.

[50] Spalding K. L., Bergmann O., Alkass K. (2013). Dynamics of hippocampal neurogenesis in adult humans. *Cell*.

[51] Braun H., Schafer K., Hollt V. (2002). BetaIII tubulin-expressing neurons reveal enhanced neurogenesis in hippocampal and cortical structures after a contusion trauma in rats. *Journal of Neurotrauma*.

[52] Urrea C., Castellanos D. A., Sagen J., Tsoulfas P., Bramlett H. M., Dietrich W. D. (2007). Widespread cellular proliferation and focal neurogenesis after traumatic brain injury in the rat. *Restorative Neurology and Neuroscience*.

[53] Sundholm-Peters N. L., Yang H. K., Goings G. E., Walker A. S., Szele F. G. (2005). Subventricular zone neuroblasts emigrate toward cortical lesions. *Journal of Neuropathology and Experimental Neurology*.

[54] Sun D., McGinn M. J., Zhou Z., Harvey H. B., Bullock M. R., Colello R. J. (2007). Anatomical integration of newly generated dentate granule neurons following traumatic brain injury in adult rats and its association to cognitive recovery. *Experimental Neurology*.

[55] Ernst A., Alkass K., Bernard S. (2014). Neurogenesis in the striatum of the adult human brain. *Cell*.

[56] Magnusson J. P., Goritz C., Tatarishvili J. (2014). A latent neurogenic program in astrocytes regulated by Notch signaling in the mouse. *Science*.

[57] Jin K., Galvan V., Xie L. (2004). Enhanced neurogenesis in Alzheimer’s disease transgenic (PDGF-APPSw,Ind) mice. *Proceedings of the National Academy of Sciences of the United States of America*.

[58] Crespel A., Rigau V., Coubes P. (2005). Increased number of neural progenitors in human temporal lobe epilepsy. *Neurobiology of Disease*.

[59] Sgubin D., Aztiria E., Perin A., Longatti P., Leanza G. (2007). Activation of endogenous neural stem cells in the adult human brain following subarachnoid hemorrhage. *Journal of Neuroscience Research*.

[60] Anderson V. A., Catroppa C., Rosenfeld J., Haritou F., Morse S. A. (2000). Recovery of memory function following traumatic brain injury in pre-school children. *Brain Injury*.

[61] Sun D., Bullock M. R., Altememi N. (2010). The effect of epidermal growth factor in the injured brain after trauma in rats. *Journal of Neurotrauma*.

[62] Sun D., Bullock M. R., McGinn M. J. (2009). Basic fibroblast growth factor-enhanced neurogenesis contributes to cognitive recovery in rats following traumatic brain injury. *Experimental Neurology*.

[63] Thau-Zuchman O., Shohami E., Alexandrovich A. G., Leker R. R. (2010). Vascular endothelial growth factor increases neurogenesis after traumatic brain injury. *Journal of Cerebral Blood Flow and Metabolism*.

[64] Lee C., Agoston D. V. (2010). Vascular endothelial growth factor is involved in mediating increased de novo hippocampal neurogenesis in response to traumatic brain injury. *Journal of Neurotrauma*.

[65] Lu D., Qu C., Goussev A. (2007). Statins increase neurogenesis in the dentate gyrus, reduce delayed neuronal death in the hippocampal CA3 region, and improve spatial learning in rat after traumatic brain injury. *Journal of Neurotrauma*.

[66] Xiong Y., Mahmood A., Meng Y. (2010). Delayed administration of erythropoietin reducing hippocampal cell loss, enhancing angiogenesis and neurogenesis, and improving functional outcome following traumatic brain injury in rats: comparison of treatment with single and triple dose. *Journal of Neurosurgery*.

[67] Han X., Tong J., Zhang J. (2011). Imipramine treatment improves cognitive outcome associated with enhanced hippocampal neurogenesis after traumatic brain injury in mice. *Journal of Neurotrauma*.

[68] Cho K. O., Lybrand Z. R., Ito N. (2015). Aberrant hippocampal neurogenesis contributes to epilepsy and associated cognitive decline. *Nature Communications*.

[69] Sun D. (2016). Endogenous neurogenic cell response in the mature mammalian brain following traumatic injury. *Experimental Neurology*.

[70] Ikeda R., Kurokawa M. S., Chiba S. (2005). Transplantation of neural cells derived from retinoic acid-treated cynomolgus monkey embryonic stem cells successfully improved motor function of hemiplegic mice with experimental brain injury. *Neurobiology of Disease*.

[71] Haus D. L., Lopez-Velazquez L., Gold E. M. (2016). Transplantation of human neural stem cells restores cognition in an immunodeficient rodent model of traumatic brain injury. *Experimental Neurology*.

[72] Peruzzaro S. T., Gallagher J., Dunkerson J. (2013). The impact of enriched environment and transplantation of murine cortical embryonic stem cells on recovery from controlled cortical contusion injury. *Restorative Neurology and Neuroscience*.

[73] Sohni A., Verfaillie C. M. (2011). Multipotent adult progenitor cells. *Best Practice & Research. Clinical Haematology*.

[74] Jiang Y., Jahagirdar B. N., Reinhardt R. L. (2002). Pluripotency of mesenchymal stem cells derived from adult marrow. *Nature*.

[75] Roobrouck V. D., Clavel C., Jacobs S. A. (2011). Differentiation potential of human postnatal mesenchymal stem cells, mesoangioblasts, and multipotent adult progenitor cells reflected in their transcriptome and partially influenced by the culture conditions. *Stem Cells*.

[76] Bedi S. S., Hetz R., Thomas C. (2013). Intravenous multipotent adult progenitor cell therapy attenuates activated microglial/macrophage response and improves spatial learning after traumatic brain injury. *Stem Cells Translational Medicine*.

[77] Samuel Dobrowolski G. L. (2013). Stem cells in traumatic brain injury. *American Journal of Neuroscience*.

[78] Ma D. K., Bonaguidi M. A., Ming G. L., Song H. (2009). Adult neural stem cells in the mammalian central nervous system. *Cell Research*.

[79] Sun D., Gugliotta M., Rolfe A. (2011). Sustained survival and maturation of adult neural stem/progenitor cells after transplantation into the injured brain. *Journal of Neurotrauma*.

[80] Harting M. T., Sloan L. E., Jimenez F., Baumgartner J., Cox C. S. (2009). Subacute neural stem cell therapy for traumatic brain injury. *The Journal of Surgical Research*.

[81] Park D., Joo S. S., Kim T. K. (2012). Human neural stem cells overexpressing choline acetyltransferase restore cognitive function of kainic acid-induced learning and memory deficit animals. *Cell Transplantation*.

[82] Lee S. T., Chu K., Jung K. H. (2008). Anti-inflammatory mechanism of intravascular neural stem cell transplantation in haemorrhagic stroke. *Brain*.

[83] Bhalala O. G., Pan L., Sahni V. (2012). microRNA-21 regulates astrocytic response following spinal cord injury. *The Journal of Neuroscience*.

[84] Walker P. A., Aroom K. R., Jimenez F. (2009). Advances in progenitor cell therapy using scaffolding constructs for central nervous system injury. *Stem Cell Reviews*.

[85] Wang J., Chao F., Han F. (2013). PET demonstrates functional recovery after transplantation of induced pluripotent stem cells in a rat model of cerebral ischemic injury. *Journal of Nuclear Medicine*.

[86] Dunkerson J., Moritz K. E., Young J. (2014). Combining enriched environment and induced pluripotent stem cell therapy results in improved cognitive and motor function following traumatic brain injury. *Restorative Neurology and Neuroscience*.

[87] Hentze H., Graichen R., Colman A. (2007). Cell therapy and the safety of embryonic stem cell-derived grafts. *Trends in Biotechnology*.

[88] Zhao Q., Gregory C. A., Lee R. H. (2015). MSCs derived from iPSCs with a modified protocol are tumor-tropic but have much less potential to promote tumors than bone marrow MSCs. *Proceedings of the National Academy of Sciences of the United States of America*.

[89] Itakura G., Kawabata S., Ando M. (2017). Fail-safe system against potential tumorigenicity after transplantation of iPSC derivatives. *Stem Cell Reports*.

[90] Yagyu S., Hoyos V., Del Bufalo F., Brenner M. K. (2015). An inducible caspase-9 suicide gene to improve the safety of therapy using human induced pluripotent stem cells. *Molecular Therapy*.

[91] da Silva Meirelles L., Chagastelles P. C., Nardi N. B. (2006). Mesenchymal stem cells reside in virtually all post-natal organs and tissues. *Journal of Cell Science*.

[92] Sanchez-Ramos J., Song S., Cardozo-Pelaez F. (2000). Adult bone marrow stromal cells differentiate into neural cells in vitro. *Experimental Neurology*.

[93] Chamberlain G., Fox J., Ashton B., Middleton J. (2007). Concise review: mesenchymal stem cells: their phenotype, differentiation capacity, immunological features, and potential for homing. *Stem Cells*.

[94] Wang S., Kan Q., Sun Y. (2013). Caveolin-1 regulates neural differentiation of rat bone mesenchymal stem cells into neurons by modulating Notch signaling. *International Journal of Developmental Neuroscience*.

[95] Ponte A. L., Marais E., Gallay N. (2007). The in vitro migration capacity of human bone marrow mesenchymal stem cells: comparison of chemokine and growth factor chemotactic activities. *Stem Cells*.

[96] Meirelles Lda S., Fontes A. M., Covas D. T., Caplan A. I. (2009). Mechanisms involved in the therapeutic properties of mesenchymal stem cells. *Cytokine & Growth Factor Reviews*.

[97] Zhang R., Liu Y., Yan K. (2013). Anti-inflammatory and immunomodulatory mechanisms of mesenchymal stem cell transplantation in experimental traumatic brain injury. *Journal of Neuroinflammation*.

[98] Bartholomew A., Sturgeon C., Siatskas M. (2002). Mesenchymal stem cells suppress lymphocyte proliferation in vitro and prolong skin graft survival in vivo. *Experimental Hematology*.

[99] Hasan A., Deeb G., Rahal R. (2017). Mesenchymal stem cells in the treatment of traumatic brain injury. *Frontiers in Neurology*.

[100] Menge T., Zhao Y., Zhao J. (2012). Mesenchymal stem cells regulate blood-brain barrier integrity through TIMP3 release after traumatic brain injury. *Science Translational Medicine*.

[101] Li L., Jiang Q., Qu C. S. (2011). Transplantation of marrow stromal cells restores cerebral blood flow and reduces cerebral atrophy in rats with traumatic brain injury: in vivo MRI study. *Journal of Neurotrauma*.

[102] Matsushita T., Kibayashi T., Katayama T. (2011). Mesenchymal stem cells transmigrate across brain microvascular endothelial cell monolayers through transiently formed inter-endothelial gaps. *Neuroscience Letters*.

[103] Lam P. K., Lo A. W., Wang K. K. (2013). Transplantation of mesenchymal stem cells to the brain by topical application in an experimental traumatic brain injury model. *Journal of Clinical Neuroscience*.

[104] Azari H. (2013). Isolation and enrichment of defined neural cell populations from heterogeneous neural stem cell progeny. *Methods in Molecular Biology*.

[105] Djouad F., Plence P., Bony C. (2003). Immunosuppressive effect of mesenchymal stem cells favors tumor growth in allogeneic animals. *Blood*.

[106] Zhang Z. X., Guan L. X., Zhang K., Zhang Q., Dai L. J. (2008). A combined procedure to deliver autologous mesenchymal stromal cells to patients with traumatic brain injury. *Cytotherapy*.

[107] Wang Z., Luo Y., Chen L., Liang W. (2017). Safety of neural stem cell transplantation in patients with severe traumatic brain injury. *Experimental and Therapeutic Medicine*.

[108] Gronthos S., Zannettino A. C., Hay S. J. (2003). Molecular and cellular characterisation of highly purified stromal stem cells derived from human bone marrow. *Journal of Cell Science*.

[109] Shen Q., Yin Y., Xia Q. J. (2016). Bone marrow stromal cells promote neuronal restoration in rats with traumatic brain injury: involvement of GDNF regulating BAD and BAX signaling. *Cellular Physiology and Biochemistry*.

[110] Cox C. S., Hetz R. A., Liao G. P. (2017). Treatment of severe adult traumatic brain injury using bone marrow mononuclear cells. *Stem Cells*.

[111] Laroni A., de Rosbo N. K., Uccelli A. (2015). Mesenchymal stem cells for the treatment of neurological diseases: immunoregulation beyond neuroprotection. *Immunology Letters*.

[112] Weiss J. N., Levy S. (2016). Neurological stem cell treatment study (NEST) using bone marrow derived stem cells for the treatment of neurological disorders and injuries: study protocol for a nonrandomized efficacy trial. *Clinical Trials in Degenerative Diseases*.

[113] Simons M., Raposo G. (2009). Exosomes–vesicular carriers for intercellular communication. *Current Opinion in Cell Biology*.

[114] Xin H., Li Y., Cui Y., Yang J. J., Zhang Z. G., Chopp M. (2013). Systemic administration of exosomes released from mesenchymal stromal cells promote functional recovery and neurovascular plasticity after stroke in rats. *Journal of Cerebral Blood Flow and Metabolism*.

[115] Zhang Z. G., Chopp M. (2016). Exosomes in stroke pathogenesis and therapy. *The Journal of Clinical Investigation*.

[116] Xin H., Li Y., Buller B. (2012). Exosome-mediated transfer of miR-133b from multipotent mesenchymal stromal cells to neural cells contributes to neurite outgrowth. *Stem Cells*.

[117] Zhang Y., Chopp M., Meng Y. (2015). Effect of exosomes derived from multipluripotent mesenchymal stromal cells on functional recovery and neurovascular plasticity in rats after traumatic brain injury. *Journal of Neurosurgery*.

[118] Zhang Y., Chopp M., Zhang Z. G. (2016). Systemic administration of cell-free exosomes generated by human bone marrow derived mesenchymal stem cells cultured under 2D and 3D conditions improves functional recovery in rats after traumatic brain injury. *Neurochemistry International*.

[119] Oh J. Y., Ko J. H., Lee H. J. (2014). Mesenchymal stem/stromal cells inhibit the NLRP3 inflammasome by decreasing mitochondrial reactive oxygen species. *Stem Cells*.

[120] Kim D. K., Nishida H., An S. Y., Shetty A. K., Bartosh T. J., Prockop D. J. (2016). Chromatographically isolated CD63+CD81+ extracellular vesicles from mesenchymal stromal cells rescue cognitive impairments after TBI. *Proceedings of the National Academy of Sciences of the United States of America*.

[121] Prockop D. J., Keating A. (2012). Relearning the lessons of genomic stability of human cells during expansion in culture: implications for clinical research. *Stem Cells*.

[122] Sekiya I., Larson B. L., Smith J. R., Pochampally R., Cui J. G., Prockop D. J. (2002). Expansion of human adult stem cells from bone marrow stroma: conditions that maximize the yields of early progenitors and evaluate their quality. *Stem Cells*.

[123] Yun Y. I., Park S. Y., Lee H. J. (2017). Comparison of the anti-inflammatory effects of induced pluripotent stem cell-derived and bone marrow-derived mesenchymal stromal cells in a murine model of corneal injury. *Cytotherapy*.

[124] Fischer U. M., Harting M. T., Jimenez F. (2009). Pulmonary passage is a major obstacle for intravenous stem cell delivery: the pulmonary first-pass effect. *Stem Cells and Development*.

[125] Walker P. A., Shah S. K., Jimenez F. (2010). Intravenous multipotent adult progenitor cell therapy for traumatic brain injury: preserving the blood brain barrier via an interaction with splenocytes. *Experimental Neurology*.

[126] Mac Donald C. L., Johnson A. M., Cooper D. (2011). Detection of blast-related traumatic brain injury in U.S. military personnel. *The New England Journal of Medicine*.

[127] Mittl R. L., Grossman R. I., Hiehle J. F. (1994). Prevalence of MR evidence of diffuse axonal injury in patients with mild head injury and normal head CT findings. *AJNR. American Journal of Neuroradiology*.

[128] Xu L., Ryu J., Hiel H. (2015). Transplantation of human oligodendrocyte progenitor cells in an animal model of diffuse traumatic axonal injury: survival and differentiation. *Stem Cell Research & Therapy*.

[129] Wang S., Cheng H., Dai G. (2013). Umbilical cord mesenchymal stem cell transplantation significantly improves neurological function in patients with sequelae of traumatic brain injury. *Brain Research*.

[130] Tian C., Wang X., Wang X. (2013). Autologous bone marrow mesenchymal stem cell therapy in the subacute stage of traumatic brain injury by lumbar puncture. *Experimental and Clinical Transplantation*.

[131] Liu L., Wei H., Chen F., Wang J., Dong J. F., Zhang J. (2011). Endothelial progenitor cells correlate with clinical outcome of traumatic brain injury. *Critical Care Medicine*.

